# Myocardial strain analysis of echocardiography based on deep learning

**DOI:** 10.3389/fcvm.2022.1067760

**Published:** 2022-12-16

**Authors:** Yinlong Deng, Peiwei Cai, Li Zhang, Xiongcheng Cao, Yequn Chen, Shiyan Jiang, Zhemin Zhuang, Bin Wang

**Affiliations:** ^1^Department of Cardiology, The First Affiliated Hospital of Shantou University Medical College, Shantou, China; ^2^Department of Preventive Medicine, Shantou University Medical College, Shantou, China; ^3^Ultrasound Division, The First Affiliated Hospital of Shantou University Medical College, Shantou, China; ^4^Department of Electronic Information Engineering, College of Engineering, Shantou University, Shantou, China

**Keywords:** echocardiography, strain, deep learning, segmentation, motion estimation

## Abstract

**Background:**

Strain analysis provides more thorough spatiotemporal signatures for myocardial contraction, which is helpful for early detection of cardiac insufficiency. The use of deep learning (DL) to automatically measure myocardial strain from echocardiogram videos has garnered recent attention. However, the development of key techniques including segmentation and motion estimation remains a challenge. In this work, we developed a novel DL-based framework for myocardial segmentation and motion estimation to generate strain measures from echocardiogram videos.

**Methods:**

Three-dimensional (3D) Convolutional Neural Network (CNN) was developed for myocardial segmentation and optical flow network for motion estimation. The segmentation network was used to define the region of interest (ROI), and the optical flow network was used to estimate the pixel motion in the ROI. We performed a model architecture search to identify the optimal base architecture for motion estimation. The final workflow design and associated hyperparameters are the result of a careful implementation. In addition, we compared the DL model with a traditional speck tracking algorithm on an independent, external clinical data. Each video was double-blind measured by an ultrasound expert and a DL expert using speck tracking echocardiography (STE) and DL method, respectively.

**Results:**

The DL method successfully performed automatic segmentation, motion estimation, and global longitudinal strain (GLS) measurements in all examinations. The 3D segmentation has better spatio-temporal smoothness, average dice correlation reaches 0.82, and the effect of target frame is better than that of previous 2D networks. The best motion estimation network achieved an average end-point error of 0.05 ± 0.03 mm per frame, better than previously reported state-of-the-art. The DL method showed no significant difference relative to the traditional method in GLS measurement, Spearman correlation of 0.90 (*p* < 0.001) and mean bias −1.2 ± 1.5%.

**Conclusion:**

In conclusion, our method exhibits better segmentation and motion estimation performance and demonstrates the feasibility of DL method for automatic strain analysis. The DL approach helps reduce time consumption and human effort, which holds great promise for translational research and precision medicine efforts.

## 1 Introduction

Assessment of cardiac mechanics has an essential role in diagnosis, risk stratification, and treatment strategies in patients with cardiac disease (1). The left ventricular ejection fraction (LVEF) is often used as a cardiac functional index, but it has a significant limitation when mechanical impairment occurs without an ejection fraction reduction (2). Alternatively, clinicians recommend finer markers of cardiac mechanical dysfunction (3). Strain imaging is richer description tool of cardiac function, which provides a more thorough characterization of myocardial contraction mechanics (4). It has applications in various cardiac pathologies (5, 6). By assessing myocardial deformation, it can detect left ventricular dysfunction before a change in LVEF.

Currently, with rapid image acquisition and relatively low cost, the most extensively utilized modality in strain imaging is two-dimensional (2D) transthoracic speck tracking echocardiography (STE) (7), which is used to estimate pixel block motion within regions along the myocardial wall (8). However, it still has several unsolved challenges due to fundamental limitations of ultrasound image modality and algorithm ad-hoc setups, including inaccurate reflection of underlying biomechanical motion and some degree of non-conclusive results caused by errors related to image quality and algorithm assumptions (9, 10). In addition, in clinical applications, there are several steps that require observer manual intervention such as view selection, adjustment of myocardial wall boundaries, and selection of tuneable parameters for tracking algorithms. This is a time-consuming and artificially introduced difference process that requires considerable expertise (10); the time spent completing a single global longitudinal strain (GLS) analysis has been found to range between 5 and 10 min (11, 12), making it inefficient in clinical practice. As a result, it is difficult to integrate into the existing cardiac ultrasound workflow, limiting the wider clinical applicability of these techniques.

Artificial intelligence (AI) advances have opened up new possibilities in this regard. Many AI-based echocardiography interpretations have been proposed in recent years, some of which are equivalent to replicating what clinicians do with a visual diagnostic rather than a more thorough analysis of what is happening with the image (13). These methods are still limited in exploring the value of ultrasound images. Meticulous quantitative evaluation is another advantage of AI, such as pixel-level segmentation and motion prediction. Automatic delineation approaches have been implemented within computational pipelines (9, 14, 15). Recent studies have shown that motion tracking also can be treated as a learnable problem and is more robust than traditional approaches (e.g., variational) in some applications (16–18). This has sparked a lot of interest in applying deep learning (DL) techniques to assess cardiac strain in echocardiography. Østvik et al. (19) have integrated cardiac view classification, event detection, myocardial segmentation, and motion estimation to construct an AI pipeline for fully automated GLS calculations. The Pwc-net (20) was used to learn to estimate ultrasonic motion, which performance was on par or better than state-of-the-art methods for traditional estimation. AI-pipeline has been shown as a promising alternative to traditional methods with significantly higher analytical efficiency. It is reported that the traditional method takes 5–10 min to measure each time, while the AI method was performed in <15 s (21). However, this method involves several sources of limited accuracy, especially the 2D segmentation and motion networks being the fundamental building blocks of the measurements.

Therefore, to improve clinical availability, we developed a more reliable DL framework for segmentation and motion estimation of echocardiogram videos to complete strain analysis. Segmentation, as a pre-step in motion estimation, is used to define the region of interest (ROI) and initialize the myocardial coordinate system for strain analysis. Previous attempts to segment left ventricular myocardium (LVM) with 2D U-net relied on manually labeled still images at end-systole (ES) and end-diastole (ED) instead of using the actual echocardiogram videos (22). These models are still limited in their ability to generalize, especially in the case of poor image quality (23). We used video instead of still frame trained three-dimensional (2D+t) CNN to segment myocardium. The three-dimensional (3D) convolution can simultaneously scan multiple frames and learn the relationships between them to constrain myocardial boundaries. Further, the optical flow CNN was employed to estimate pixel motion, which is the task of estimating the instantaneous velocity of pixels in the ROI (24). In previous studies, optical flow CNN for cardiac motion estimation from echocardiography has been demonstrated to be feasible, like EchoPwc-net and Flownet (19, 25). But the best systems are still limited by difficulties including fast movement, occlusions, and motion blur. To address these deficiencies, we attempted a new motion estimation method and compared it with Pwc-net and Flownet. RAFT is a new motion estimation model proposed in recent years (24). The algorithm uses additional recurrent neural network to optimize motion details, and has better performance in fast motion and occlusion. In our work, we proposed a 3D network for echocardiographic myocardial segmentation and compared several classical motion estimation algorithms to achieve global and local strain analysis.

Therefore, in this study, we constructed a new fast, fully automatic myocardial strain analysis workflow consisting of segmentation and motion estimation convolutional neural networks. This approach provides visually assessable tracings of the myocardial motion, which facilitate human assessment and downstream analysis. And that, the accuracy and repeatability of the proposed framework are verified *in vivo* data, which is critical for clinical adoption (10). Compared with previous studies, our model has more potential in automatic quantitative analysis of echocardiography. This could make the measurements more robust and hopefully replace traditional methods to achieve automatic strain measurement without observer intervention, helping to improve clinical workflow.

## 2 Materials and methods

### 2.1 The architecture of deep learning model

**FIGURE 1:**
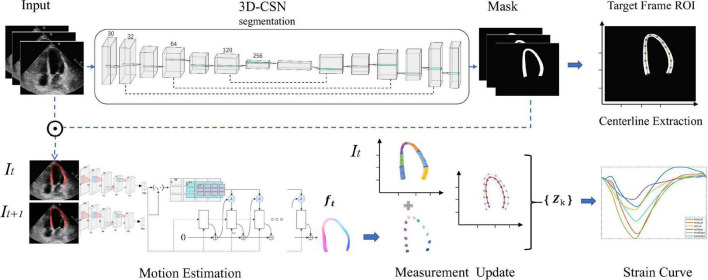
Overview of the deep learning (DL) workflow for automatic measurement of longitudinal strain. 3D-CSN uses multi-frame echocardiogram images as input to delineate the region of interest and segments, and extract the centerline of target frame. Then, the motion network estimates the movement field of the pixels in the ROI to generate the velocity vector at each moment. These velocities are used to propagate the position of the centerline and then calculate the global and local line arc lengths coexisting in {Zk} which are used as a basis for strain measurements.

We construct a DL workflow for cardiac segmentation, motion estimation, and calculating GLS, which has three key components (Figure 1). Let *I_t_* be a frame at time *t*.

### 2.2 Segmentation and LVM localization

First, we developed a 3D cardiac segmentation network (3D-CSN) with U-net type for frame-level semantic segmentation of the cardiac to generate ROI and establish *I_0_* LVM coordinate system for strain analysis. The segmentation network is a 3D architecture that uses a 3D array with size 256 × 256 × n (256 × 256 represents the image size, and n represent the number of frames) to generate a segmentation mask of equal size, each color corresponding to a tissue label (i.e., background and LVM). It has an encoder and a decoder path each with four layers. In the encoder path, each layer contains two 3D convolutions (3 × 3 × 3) each followed by a rectified linear unit (ReLu) and max pooling (2 × 2 × 2). In the decoder path, each layer consists of an upconvolution (2 × 2 × 2) followed by two 3D convolutions each followed by a ReLu. The kernel of 3D convolution allows the model to simultaneously scan three successive frames and learn spatial features in two dimensions (x, y) as well as temporal information in the third dimension (*t*), which has previously performed well in video classification tasks (26). The encoder path is used to learn video spatiotemporal features, and then generate the same resolution mask through decoder path. Skip connection is used to propagate details. Thus, 3D-CSN uses a 3-channel input volume composed of 3 consecutive frames of images to generate a 3-channel array o of equal size, each channel representing the mask of each frame. The target image and mask are overlapped to obtain the segmentation result, namely the ROI (Ω). Skeleton extraction algorithm is then employed to take the centerline (_ʗ_⊆Ω) between the endocardium and the epicardium on end-diastole (*I_0_*), which will be used to locate the myocardium and generate coordinates. The centerline, defined as the mid-point between two nearest endo- and epicardial points, was used to track and calculate myocardial strain.

### 2.3 Motion estimation

Second, we constructed an optical-flow based motion estimation network used to estimate each pixel (v∈Ω) movement field (*f_t_*) of the heart from *I_t_* to *I*_*t+1*_, which is used to update the position of LVM centerline (ʗ). In this study, we employ three different variants (RAFT, Pwc-net, and Flownet) to identify the optimum basic architecture and eventually chose RAFT as the best performing architecture. RAFT, based on an optimization approach, consists of three main components: feature encoder, correlation layer, and update operator. This method involves taking two consecutive images (*I_t_*, *I*_*t+1*_) as input, and these are fed separately into feature encoders with shared weights. The feature encoder contains six residual blocks and is downsampled three times; the amount of filters successively is 64,64,128,128, and 192,192. After feature extraction, the block maps the input images to dense feature maps (*g*θ) at 1/8 resolution. Then we compute visual similarity by constructing the dot product between all pairs of feature vectors. It can be efficiently computed as single matrix multiplication


(1) $$C_{i\,j\,k\,l}=\sum_{h}g\,\theta \,{\left(I_{1}\right)}_{i\,j\,h}.g\,\theta \,{\left(I_{2}\right)}_{k\,l\,h}$$

Where *C*_*ijkl*_ is the correlation layer, *ij* and *kl* represent pixel coordinates of *g*θ(*I*_1_) and *g*θ(*I*_2_), respectively, and h denotes the channel of feature maps.

The correlation volume *C*_*ijkl*_ contains four correlation layers of different sizes by pooling *kl* dimensions with kernel sizes 1, 2, 4, and 8 strides, which stored both large and small displacements information. Meanwhile, it maintains high-resolution information by maintaining the *ij* dimensions, allowing this method could recover the motions of small fast-moving objects. Finally, the network uses the current flow field *f_k_* to retrieve correlation features *C* from the correlation layer and then concatenate them to input ConvGRU-based (27) update operator that produces an update direction Δ*f* to update *f_k_*, *f_k_* is initially set to zero (**f_0_** = **0**). Update operator is a lightweight network based on recurrent neural network, which can set any number of iterations to optimize the flow field. After 12 iterations, we decided on the final optical flow. Thus, RAFT uses two consecutive 1-channel input images with size 256 × 256 to generate a 2-channel array *f* of equal size, each channel representing the x and y components of motion, which is used to update the x and y coordinates of LVM pixels, respectively. Structural comparisons of RAFT, Pwc-net, and Flownet are discussed in Supplementary section 1.

### 2.4 Tracking update and calculate strain

Finally, we update the position of myocardial centerline _ʗ_ by displacement field *f*, i.e., _ʗ_(*t* + 1) = _ʗ_(*t*)+ *f*(*t*), and calculate its arclength τ which represents the longitudinal length of LVM at each moment. Strain represents percent change in myocardial fibers length per unit under stress (13). Thus τ is used to estimate GLS


(2) ς⁢(t)=[τ⁢(t)-τ⁢(0)]/τ⁢(0)

**FIGURE 2:**
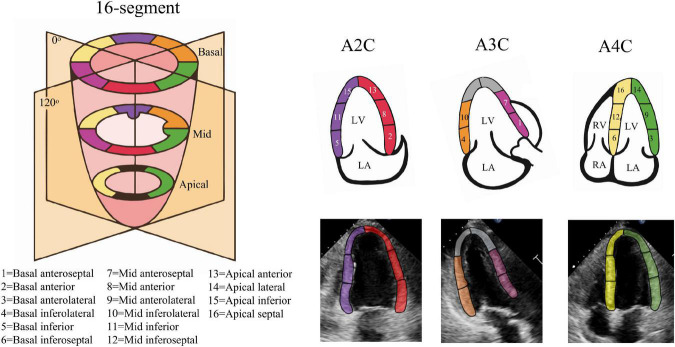
Echocardiography 16-segment. The 2015 ASE guideline recommend that the left ventricular myocardium be divided into three rings, namely the basal ring, the mid ring, and the apical ring, each with a height 1/3 of the length of the left ventricle. The basal ring and the mid ring contain six segments, respectively, and the apical ring contains four segments. The actual echocardiogram corresponds to the diagram above. The colors indicate different supplying coronary arteries.

Where τ(*t*) denotes ventricular longitudinal length at frame *t*, τ(0) is ED frame ventricular longitudinal length. The peak-GLS was defined as the minima strain value within a cardiac cycle. For segments, we adopt 16-segment division method (Figure 2), with the apex of ED centerline as the demarcation node, divided three arcs of the same size on both sides as the initial position of each segment (28), and calculated regional longitudinal strain (RLS) in subsequent updates.

## 3 Experiments

### 3.1 Data preparation

The echocardiography dataset consists of two parts, one for model training and the other for external clinical validation.

**FIGURE 3:**
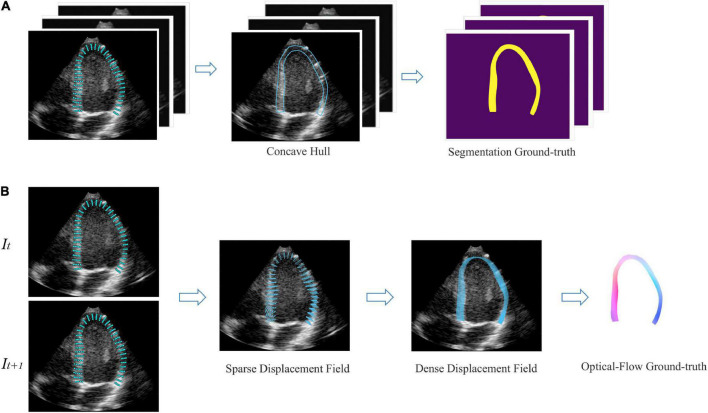
Semi-automatic data annotation. **(A)** Segmentation marked, the concave hull formed by connecting the outermost point is marked as the LVM region. All pixels within the hull are marked as “1,” as shown in yellow, and those outside are marked as “0,” as shown in purple. Every three frames as a data. **(B)** Optical flow marked, *I_t_*, *I*_*t+1*_ represent two adjacent frames. The sparse displacement field is calculated according to the position changes of the corresponding points of the before and after frames. Then, the density velocity field of each pixel is generated by cubic interpolation, which is represented as flow field. Color and saturation indicate direction and magnitude, respectively.

For development, we used a publicly available dataset (29) of simulated echocardiography images, consisting of 105 sequences or 6,165 frames with apical 2-chamber (A2C), apical 3-chamber (A3C), and apical 4-chamber (A4C) views. The data is created with a complex biomechanical model for comparison of speck tracking imaging algorithms. Data templates come from seven different vendors and five motion patterns, including one healthy and four pathologies. For each frame, the authors provide a set of 180 points coordinate *P*_*k*_ <x, y>, *k* {1,2…180} evenly distributed in the myocardial region, corresponding to the underlying motion field of the LV myocardium. Therefore, semi-automatic labeling was employed to generate labels for monitoring network training. As shown in Figure 3A, we annotate the region of LVM in each frame by a concave hull that connects the peripheral points, medial line is defined as the endocardium and the lateral line as the epicardium. Inside the myocardium labels “1,” others label “0.” Every three consecutive frames as a data is converted to a 3D array, with the corresponding label as input. In order to generalize the model in reality, we additionally fine-tuned the model on 200 manually annotated datasets. For motion estimation network, we used the coordinate changes of corresponding points between consecutive frames to generate a sparse displacement field, i.e., *V_k_ = P_k_* (t + 1) − *P_k_* (t), and then used cubic interpolation to convert it to a dense displacement map with velocities inside the myocardium as shown in Figure 3B. The dense Displacement map is used as the ground truth flow to monitor gradient descent of the motion network. In order to expand the range of motion amplitude distribution between frames, we used sampling every other frame to clip the raw video. Each video was cut into 4 cine-loop. Finally, 4200 fully labeled echocardiographic data were generated for 3D-CSN training, and 12,000 ultrasound image pairs for the development of the motion network. For the robustness of model, data augmentation including basic augmentations and ultrasound-specific augmentation routines was applied (19). Details of data edit and enhancement are provided in Supplementary section 2.

Additionally, we evaluated the actual effectiveness of the model on an external test dataset of 150 echocardiogram videos from 50 patients from an independent hospital system (The First Affiliated Hospital of Shantou University Medical College) and in comparison with commerce STE. Three standard apical views were extracted from each patient. To ensure a representative range of LV pathologies, we included three pre-defined patient groups (Supplementary section 3): 17 patients with myocardial infarction, 17 patients with ischemic heart failure, and 16 patients admitted for chest pain without any evidence of cardiac origin. Videos were acquired by skilled sonographers using PHILIPS Epiq 7C ultrasound machine and processed images were stored in a Philips Xcelera picture archiving and communication system. The study was approved by the Regional Committee for Medical and Health Research Ethics (No. B-2022-196) and all patients gave written consent. Patients were included consecutively for each group regardless of image quality. Exclusion criteria were significant ventricular aneurysm, atrial fibrillation, age younger than 18 years, or inability to give written informed consent. Supplementary Table 1 summarizes the characteristics of the included patients.

### 3.2 Model development and training

Model design and training were done in Python using the PyTorch deep learning library. The hardware consisted of an Intel Core CPU i7-11700k, 32 GB RAM, and an NVIDIA RTX3090 GPU with 24 GB of memory. The training process is divided into two sections: The 3D semantic segmentation network training and motion estimation network training. All data are randomly divided into training set, validation set and test set in 7:2:1 order. Model development adopts transfer learning strategy. Segmentation network was pre-trained on simulated ultrasonic data, then fine-tuned on hand-labeled data. Motion estimation network was pre-trained on FlyingThings, then fine-tuned on the abovementioned ultrasonic data.

The essence of segmentation is pixel classification, means that every pixel on the image needs to be assigned to the target region (30). In other words, the task of 3D-CSN is to classify every pixel on the video volume into LVM or background. Model input is a 256 × 256 × 3 voxel tile together with a binary value indicating classification of each pixel,256 × 256 represents the space size of the image, and 3 indicates the number of frames. The output is a mask of the same size as the input video, where each pixel is classified as either LVM or background. To enable training of 3D network we used the memory efficient cuDNN convolution layer implementation. The model was initialized with random weights and was trained using the stochastic gradient descent with momentum (SGDM) optimizer. We set batch size of 6 and learning rate of 2e-4 for all experiments. We ran 50k and 10k iterations on pre-trained and fine-tuned, respectively, which took approximately 25 h. Different from previous Dice loss in 2D segmentation, this network output and ground truth are compared using Dice loss + Cross entropy


(3) L⁢(Y,Y′)=-1N⁢∑c=1C∑n=1N(yn,c⁢l⁢o⁢g⁢Py′n,c+2⁢|yn,c⁢⋂⁢y′n,c||yn,c|+|y|′n,c)

Where, *y*_*n,c*_∈*Y* and y′_n,c_∈*Y*′ are the target label and predictionof class *C* and *N*th in batch processing, respectively, *Y* and *Y*′ are the truth value and prediction result of input image, and *C* and *N* represent the number of classes and pixels in the dataset. P represent probability.

The model-predicted and labels were compared using Dice Similarity Coefficient (DSC) metrics at ED, ES, and random other frames. We examine the model’s segmentation effect on three views and compare it with the 2D segmentation model involved in the pipeline proposed by Smistad et al. (31). Note that 3D-CSN is trained on the data set we developed, while the 2D network is trained on CAMUS (32) data set (including the tagged ED and ES frames).

Optical flow network was initialized with pre-trained weights from the FlyingThings dataset, then fine-tuned on ultrasonic data. The model’s input is the segmentation network’s output, and the output is the dense displacement field of ultrasonic specks in two consecutive frames. We tested three different model architectures. All networks were trained with AdamW optimizer parameters beta 1, 2 = 0.9, 0.999, random initialization, and the initial learning rate = 2e-4. For fine-tuning, the initial learning rate was set to 1e-4. We pre-trained on FlyingThings for 100k iterations with a batch size of 12 and then fine-tuned on echocardiography for an additional 20k steps with a batch size of 6. Training time was approximately 2–3 days. The L2 distance between the predicted and ground truth flow was used to supervise network training. The loss is defined as


(4) $$L=\sum_{i=1}^{N}\gamma^{I-i}\,{||f_{g\,t}-f_{i}||}^{2}$$

Where *N* represents iteration times, *f*_*gt*_ stand for flow ground truth, *f_i_* represents the *i*th predicted flow, γ = 0.8.

The accuracy was assessed using the in-plane end-point error (EPE) between predicted V’ and ground truth *V*, it is defined as the Euclidean distance between the ground truth velocity and the predictions.


(5) $$E\,P\,E\,\left(v,v^{\prime }\right)={\sqrt{{\left(V_{x}-V_{x}^{\prime }\right)}^{2}+\left(V_{y}-V_{y}^{\prime }\right)}}^{2}$$

Finally, our model tracked the position of the centerline frame by frame based on the optical flow field and used this as the longitudinal length of the myocardium. Then the strain value at each moment was calculated according to formula (2), and finally formed the myocardial strain curve.

### 3.3 Prospective clinical validation

A comparison study was performed by analyzing 150 echocardiogram videos to compare the proposed DL method and actual clinical practice measurement. We used this model and STE to test the exact same echocardiogram simultaneously, resulting in 2 paired GLS measurements and RLS value. GLS was calculated as the average peak strain of the 3 apical views. A single heart cycle (start at ED) was chosen from each view and the exact same recording and cardiac cycle was used for both methods. ED was defined by the automatic ECG trigger algorithm of the analysis software. The first measurement system consisted of a single experienced observer using a commercially available speck-tracking analyses method (Philips auto-CMQ) for strain measurements. The observer manually corrected the ROI by visual assessment of the endocardial and epicardial borders. Block matching was used for motion tracking. Spatial and temporal smoothing were kept at default values. Drift compensation was applied as by default. The speck tracking analyses were performed in accordance with the consensus document of the EACVI/ASE/Industry Task Force to standardize deformation imaging (28). The second measurement system was the DL method measuring strain without any observer intervention. The model automatically performs ROI identification, tissue division, motion estimation, and strain calculation. Analyses were performed without knowledge of clinical data or previous measurement results. Finally, to assess whether the consistency between the two methods was affected by LV pathologic type and image view, subgroup analysis of 150 echocardiographic videos was performed, classified by LV pathologic type (normal, myocardial infarction, and heart failure) and image view (A2C, A3C, or A4C).

### 3.4 Statistical analysis

Continuous variables were presented as mean ± standard deviation, and dichotomous data were presented as numbers (percentages). Association between methods was estimated by calculating the Pearson correlation coefficient. Bland–Altman (B-A) analysis and intra-group consistency comparison (ICC) was used to assess the agreement of measurement pairs. The mean absolute difference between the two measurement systems was calculated using the mean value of the absolute difference between all measurement pairs, and bias denotes the mean difference was calculated using the mean value of the difference between all measurement pairs. Tests for normality were performed using Shapiro–Wilk and Kolmogorov–Smirnov tests. ANOVA was used to assess if there was a statistically significant difference in bias between subgroups of measurement pairs when categorized using view and pathological pattern, or Brown-Forsythe test when the variance is not uniform. All statistical analyses were performed using SPSS 25.0 software (SPSS Inc, Chicago, IL, USA).

## 4 Results

### 4.1 Model for segmentation

**FIGURE 4:**
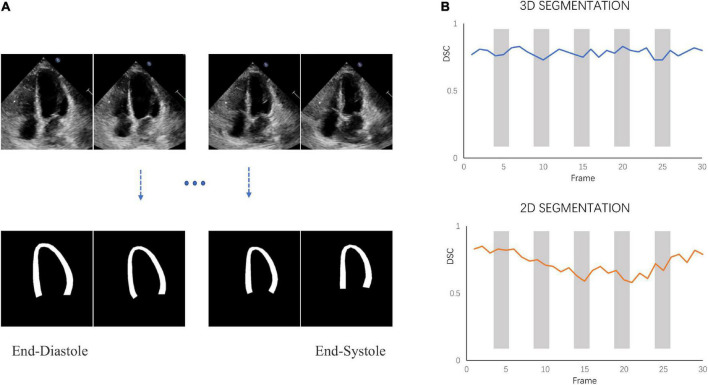
Segmentation performance. **(A)** 3D-CSN allows simultaneous segmentation of multiple frames. Inputting echocardiogram video sequence frames (top row) will generate corresponding segmentation masks (bottom row). **(B)** The dice similarity coefficient (DSC) was calculated for each frame of video on different models.

3D-CSN was trained to generate frame-level segmentations for the entire video (Figure 4A). The DSC of LVM was measured and collected in Table 1. For comparison, DSC obtained with the 2D network proposed by Smistad et al. (31) is also included in the table. With this model, the correlation of 3D-CSN measures and LVM labels was >0.79 across all measures, and the average DSC for A2C/A3C/A4C was 0.826/0.808/0.833, respectively. Further, the average DSC of 2D segmentation was 0.787/0.720/0.798. The 2D and 3D networks have achieved comparable results on ED and ES frames, but 2D network significantly worse than 3D-CSN (i.e., A3C: other 0.803 vs. 0.581) on other frames. The 3D model showed similar performance on each frame of the sequence, while the 2D model performed well on ED/ES but poorly on intermediate sequences (Figure 4B). Both 3D and 2D models perform better on the A4C plane.

**TABLE 1:** State-of-the-art method for left-ventricular myocardial segmentation shown at end-diastole (ED), end-systole (ES), and one of other sequence frame compared to 3D-CSN.

Method	A2C Dice			Avg.	A3C Dice			Avg.	A4C Dice			Avg.
	ED	ES	Other		ED	ES	Other		ED	ES	Other	
3D-CSN	0.836	0.810	0.832	0.826	0.823	0.799	0.803	0.808	0.856	0.829	0.814	0.833
Smistad et al. (31)	0.802	0.857	0.703	0.787	0.786	0.794	0.581	0.720	0.811	0.839	0.694	0.798

Avg., average.

### 4.2 Model for motion estimation

**FIGURE 5:**
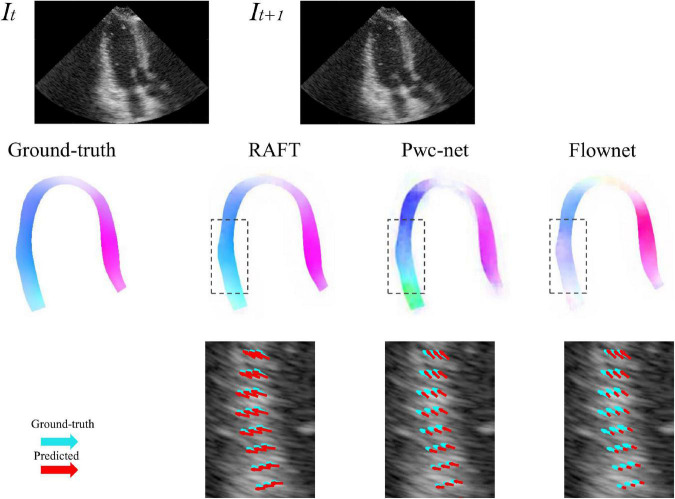
Example of predicted optical flow patterns for the different models within the myocardium. The **top row** is the sequence of input to motion estimation networks. The **middle row** is the prediction of networks, represented by color coded with hue values, color and saturation indicate direction and magnitude, respectively. The **bottom row** shows the velocity vector comparison between ground truth and the different methods inside the locality as indicated by the dotted box in the ultrasound image. Light green arrows are ground truth, while red arrows are predictions.

Five-fold cross-validation was performed on the simulated ultrasonic data, this resulted in five training sessions for each network. The average EPE (AEPE) with corresponding standard deviation can be seen in Table 2. The smallest AEPE session as the best model, RAFT/Pwc-net/Flownet is 0.05/0.08/0.12. RAFT had lower AEPE relative to Pwc-net and Flownet, and Pwc-net was better than Flownet (*p* < 0.001). Figure 5 illustrates a representative example of dense flow results for the different methods and the end-point distance between ground-truth and prediction of displacement field.

**TABLE 2:** Average end point error (AEPE) on simulated ultrasound data.

Method	AEPE [mm]				
	Fold 1	Fold 2	Fold 3	Fold 4	Fold 5
RAFT	0.05 ± 0.04	0.06 ± 0.03	**0.05 ± 0.03**	0.07 ± 0.05	0.07 ± 0.03
Pwc-net	0.10 ± 0.07	0.09 ± 0.05	0.12 ± 0.09	**0.08 ± 0.06**	0.10 ± 0.08
Flownet	0.15 ± 0.10	**0.12 ± 0.08**	0.13 ± 0.08	0.14 ± 0.10	0.13 ± 0.12

Units given in mm per timestep/frame ΔT – 1. Data are presented as mean ± standard deviation. Bold font are the best results for each model.

### 4.3 Tracking update and clinical validation

**FIGURE 6:**
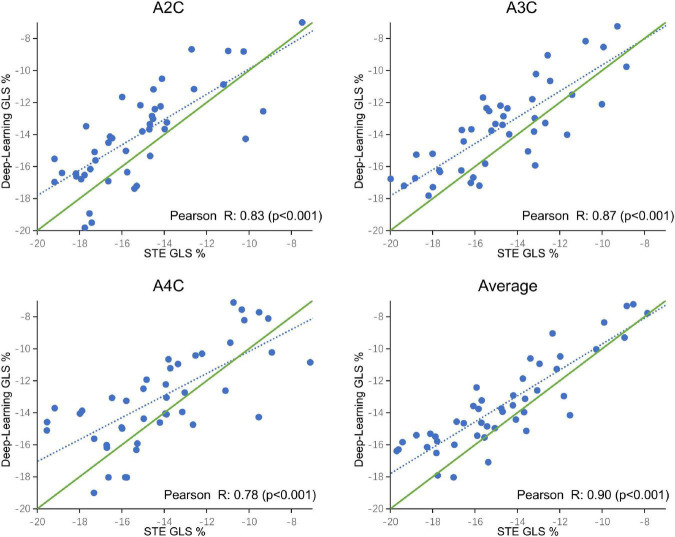
Correlation plot of global longitudinal strain (GLS) estimated between STE and deep learning-based method for specific views and averaged over the three apical views. Each dot represents one examination. Blue dotted line represents the best fit line to the data by linear regression. Green solid line represents the theoretical perfect correlation.

**FIGURE 7:**
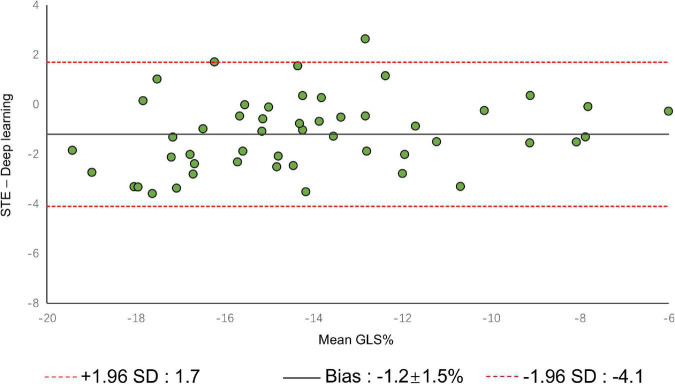
Bland–Altman plot presents the comparison of measurements between the reference method and the DL method. The figure shows the limits of agreement (LOA) of 1.96 SD (red dotted line). SD, standard deviation.

**FIGURE 8:**
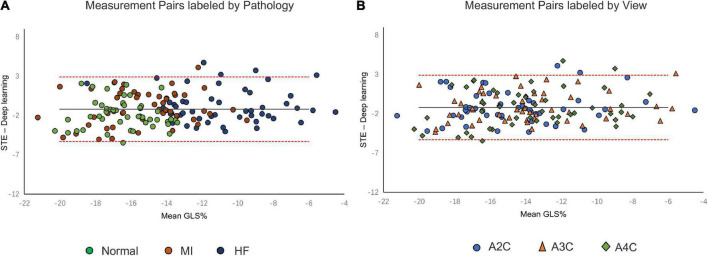
Bland–Altman plot presents the comparison of measurements between the reference method and the DL method. **(A)** Measurement pairs labeled by pathology, **(B)** and specific views. MI, myocardial infarction; IHF, ischemic heart failure.

A comparison of tracking throughout the whole cardiac cycle from a simulated ultrasonic subject is shown in Supplementary Video 1, together with the formation of GLS curves. For Clinic validation, GLS was obtained successfully in all patients. A correlation plot of the peak-GLS measured by proposed method and reference method for each individual view and the average is given in Figure 6, and correlations were 0.83, 0.87, 0.78, and 0.90, respectively. The average peak-GLS on all subjects was −13.53 ± 3.04% and −14.72 ± 3.39% for the DL method and reference method (Table 3). The mean absolute difference was 1.6 ± 1.1%. The Bland-Altman analysis of between method differences revealed a bias of −1.2 ± 1.5% (*p* < 0.01) with estimated limits of agreement (LOA) of −4.1 to 1.7% (Figure 7). There is no significant difference in bias between DL method and reference method among subgroups classified by view (*p* = 0.86). Moreover, no significant difference in bias was found between subgroups when categorized using pathological patterns (*p* = 0.07). The consistency analysis of measurement results differences between subgroups is presented in Figures 8A,B. The intra-group consistency comparison (ICC) of RLS of the 16 segments contained in the 3 views is shown in Supplementary Table 2. RLS showed good consistency only in the basal anterolateral, mid anterolateral and apical anterior, with ICC exceeding 0.8, and large instability in other segments. The bull’s eye plots display the RLS value of 16 segments measured by STE and DL method for different subjects (Supplementary Figure 3). In healthy subject, strain values in the polar map have a similar distribution. In MI patient, both maps indicate a focal strain reduction at the lower right, and inspection of the myocardium on the echocardiography shows an inferolateral infarct that coincides in location with segments with more prominent decreases in strain. In HF patient, both maps indicate a diffused strain reduction.

**TABLE 3:** Mean global longitudinal strain (GLS) for each view measured by deep learning method and reference method (standard deviation), and averaged over the three apical views.

Method	A2C GLS	A3C GLS	A4C GLS	Average
DL-method	−14.06 ± 3.36%	−13.29 ± 3.57%	−13.24 ± 3.28%	−13.53 ± 3.04%
Reference-method	−15.26 ± 3.35%	−14.43 ± 3.81%	−14.47 ± 3.73%	−14.72 ± 3.39%

The computational timing of the proposed method is approximately 5 s per video for myocardial segmentation and 100 ms per frame for motion estimation on a GPU of a standard desktop computer. Total processing time when running the entire workflow was 13 ± 2 s per view and 40 ± 5 s for a full patient analysis including all three apical views. On the same echocardiography, STE analysis was completed within 3 min per video.

## 5 Discussion

Our study describes a carefully designed automatic strain quantification DL workflow that consists of 3D segmentation and optical flow network for handling the challenges associated with echocardiographic motion tracking. It was able to determine myocardial borders, estimate motion, and ultimately compute strain. Based on tracking movement of centerline initialized from myocardial segmentation at the first frame, the DL approach measured strain frame by frame. The tracking was performed by using the displacement fields from the motion estimation network to update the position of points on the centerline. We benchmarked its segmentation, motion, and strain estimation components against the state-of-the-art. We compared our segmentation and motion estimation to other DL methods, and strain measures to a reference speck tracking technique.

3D-CSN was designed to execute semantic segmentation for determining the ROI of motion estimation network and initializing LVM position. Noise is a long-standing difficulty in the field of motion estimation in echocardiography (33). We mask the ultrasound image to remove redundant input signals and determine the LVM wall boundary. A 2D segmentation network was utilized to determine the LVM wall in Østvik et al.’s study (19), which was trained only on ED and ES frames but segmented each frame of echocardiogram video. Its segmentation is separate, ignoring the temporal and spatial continuity of myocardial movement. This result in model performed well on ED and ES frames but not on intermediate frames. In this investigation, we employed U-net with 3D convolution (3D-CSN) to complete this challenge. In all views, 3D-CSN exhibited a higher average dice score in segmenting regions and performed similarly well on all frames of a cardiac cycle. Both models were based on U-net framework, the main difference is 3D vs. 2D convolution. Two-dimensional convolution is a 3x3 block that can only scan one image at a time to learn spatial information of the image, whereas 3D convolution is a 3×3×3 volume that could scan multiple images simultaneously to learn the spatial features and inter-frame relationship of the image. Myocardial motion is regular, and when multiple frames are segmented at once, the border of the target frame will be restricted by the preceding and following frames, thus the 3D-CSN segmentation results are more spatio-temporal smoothness. In addition, DL is a typical data-driven model, the range of data is closely related to the generalization performance (4). The 3D-CSN is trained within all sequence frames, so its generalization ability is stronger. Furthermore, we believe that this design is more consistent with biological characteristics. In echocardiography, even experienced ultrasound experts sometimes have difficulty in accurately identifying the endocardium and epicardium in a single image frame, especially in the image with significant noise and artifacts, usually by repeatedly viewing the video. Therefore, 3D-CSN, similar to multi-frame observation, can infer the position of the endo- and epicardium of current frame from adjacent frames, which seems helpful for reducing the influence of image quality on segmentation model. To our knowledge, 3D-CSN is the first 3D U-net-based DL model for echocardiogram segmentation which could learn the morphological features of all frames and its performance better than that of previous image-based 2D networks. Motion estimation is another crucial part of quantifying myocardial deformation. We compared three different network structures. Table 2 suggests that the motion estimation method producing best results is RAFT, and Pwc-net is superior to Flownet. The qualitative results in Figure 5 further suggest that RAFT has a better match in velocity vectors. In recent years, optical flow network has experienced the development from encoder-decoder architecture to spatial pyramid structure and then to optimization-based network. All three kinds of networks have outstanding performance in motion estimation, but which is most suitable for echocardiography has not been determined. Østvik et al. (19, 25) have described the applications of Pwc-net and Flownet in myocardial movement, where Pwc-net outperforms Flownet, which was in line with our study due to Pwc-net introducing a refinement mechanism that optimizes flow prediction based on the pyramidal layers. However, recent reports suggest that spatial pyramid architecture may ignore small, fast-moving motions. Pwc-net adopts a coarse-to-fine strategy to refine the flow, that is, the optical flow is initialized at the minimum resolution first and then refined in the direction of high resolution, hence named pyramid architecture. This structure may result in fast-moving small objects being missed in low-resolution and challenging to recover in later iterations. Due to the lower displacement magnitudes between frames in echocardiography compared to other film actions (19), we question the validity of this architecture. In our study, we compared the RAFT and ultimately chose it as a component for motion estimation due to its excellent performance in minor motion and occlusion (34, 35). In synthetic echocardiography, the AEPE of RAFT is significantly less than Pwc-net (Table 2). Unlike Pwc-net, RAFT eschews the pyramidal refinement structure, instead performing a update operator consisting of recurrent neural network to refine optical flow by generating an unlimited number of iterations at the same resolution, which could integrate last flow deviation to optimize the current flow prediction. We set up 12 iterations and did it at a single high resolution so that the slight motion of the first iteration could also be transmitted to the last layer. Similar to the human eye, people lack an intuitive understanding of some details of movement due to the limited field of view at the low resolution. Whereas RAFT is like multiple observations at the exact resolution, repeatedly reinforcing the details. By comparison, RAFT’s specific architecture is more compliant with echocardiographic motion. We believe that further changes based on RAFT will become the new benchmark.

Therefore, our design is more in line with the biological characteristics of human experts. The LVM borders is identified by dynamic observation and eliminates the interference of noise from other regions to motion estimation. Then, the movement of each pixel was optimized for multiple iterations at a single high resolution instead of coarse-to-fine. In both the segmentation and motion estimation, our models are on par or better than the state-of-the-art.

The DL method successfully completed all GLS measurements and has a high level of agreement with currently accepted speck tracking techniques. Compared to semi-automatic cardiac motion quantification (CMQ) in Phillips ultrasound, the average GLS were −13.53 ± 3.04% and −14.72 ± 3.39%, respectively, showing an excellent correlation that achieved 0.90 (*p* < 0.001), and consistency analysis exhibited bias of −1.2% with LOA of ±2.9%. A study by Salte et al. (21) compared the previously mentioned computing pipeline with traditional methods. They reported bias of −1.4% and LOA of ±3.7%. Our results are within that range. Assessing subgroups categorized by view and pathology, there was also no statistically significant difference between DL and reference methods, suggesting that different views or pathological motion states had limited influence on consistency. The strain measured by traditional STE is not the gold standard, so we cannot determine which method is more accurate, but it is still sufficient to demonstrate the accuracy and repeatability of DL automatic strain calculation. Nevertheless, there is a large variability in regional strain, which is expected because the two methods do not achieve uniform anatomical constraints, hence there are differences in the definition of segments. However, DL method is still sensitive to the changes of local strain under pathological conditions. As with STE, the bull’s eye map (Supplementary Figure 3) from DL shows reduced strain that coincides with infarct segments in patients with infarction and reduced diffuse strain in patients with heart failure, suggesting that the DL approach is also diagnostic for myocardial disease. The currently most widely used semi-automatic speck tracking method is time-consuming and demands expertise. It involves several steps of operator intervention, such as view selection, ROI adjustment, and tunable parameter setting. Therefore, it is difficult to integrate into the existing workflow of ultrasonic cardiogram, which is mainly completed by offline analysis. The measurement accuracy of DL is comparable to or better than that of traditional methods, and the efficiency is significantly improved. With only one GPU, the DL method completes these tasks in real-time; each prediction task takes <15 s and is much more rapid than the STE assessment of myocardial strain. The rapidity and automaticity of AI greatly decrease the labor of cardiac function assessment and experiential needs. This provides the opportunity for more-frequent, rapid evaluations of cardiac strain (e.g., on-screen view strain real-time during image acquisition and application in portable ultrasound). DL methods could potentially aid clinicians with a more precise and rapid assessment of cardiac strain and early detect abnormal ventricular wall movement. In settings in which the sensitive detection of change in cardiac function is critical, early detection of change can substantially affect clinical care (36, 37).

It is worth noting that in our tests, we found that tracking may fail with poor image quality, as Salte et al. (21) reported. Poor image quality in echocardiography is a common problem that leads to invalid measurements. However, most previous DL studies have no effective monitoring means, and the primary limitation of AI in clinical application is that the internal mechanism is unclear. Hence, it is difficult for clinicians to trust it directly. Our method is different from the past’s black-box approach proposed by some authors, such as directly predicting LVEF or GLS from images (38, 39). AI models should be designed to provide visual feedback that can be checked manually. As a result, offering a visual feedback system is a vital assurance of the results’ trustworthiness. The method presented in this study was able to visually inspect if segmentation and motion estimates seem reasonable by visualizing myocardial segmentation and centerline movement (Supplementary Video 2). So when the tracking fails, it can be clearly sensed by the observer.

Our work provides a more accurate and robust scheme for automated GLS analysis and is the first model to realize local strain analysis on echocardiography. Compared with the previous strain analysis models, we propose improvements to segmentation and motion estimation. All strain measurements are successfully completed, and the results are better than previous advanced methods. This approach is expected to replace STE for real-time strain calculation in cardiac ultrasound practice. Thus, if these DL methods are integrated into a generic AI pipeline, the individual steps could be computed during the acquisition of images, allowing for rapid bedside analysis and even real-time measurements on the ultrasound scanner.

### 5.1 Study limitations

This study of DL applied to echocardiographic data has several limitations. First, the learning-based optical flow method has high precision and efficient reasoning ability. However, obtaining training data in reality is difficult, so most supervised methods heavily rely on large-scale synthetic data sets. The training data used in this study came from the processing of data synthesized according to the biological template and we are not clear about the difference between this data and real *in vivo* data. Whether training data will lead model preference remains to be further investigation. Second, DL is a method based on data-driven, which does not have reasoning ability. It tends to represent the distribution of training data. Data regional differences lead to potential degradation when models are transferred to the real world. Further, as the template of simulated data is equal across categories, we also suspect that the motion model is slightly over-fitting. We believe further optimizations can be made including real-world data acquisition and self-supervised model training. Finally, for external validation, we only compared measurements against one commerce method and lacked the gold standard metric. Thus, we cannot conclude whether the method in this study is more accurate than the traditional STE method. We could only conclude that there is a high degree of agreement of GLS measurements between the two measurement systems, while the regional strain estimates still have large variability and need to be further optimized. Due to the limited sample size, the conclusions of comparative studies may not be generalizable. Therefore, this part of the work should be considered as a pilot study for clinical comparison, further studies with cardiac magnetic resonance as the gold standard and larger sample sizes should be carried out.

## 6 Conclusion

Fully automated strain measurements based on DL have the potential to both reduce manual intervention and improve reproducibility, and, due to the processing speed of learning-based algorithms, this could eventually enable on-screen measurements in real-time while the operator acquires images. Our carefully designed structure is state-of-the-art, making the workflow an excellent candidate for use in routine clinical studies or data-driven research. In future studies, we will further optimize it to achieve robust multi-dimensional strain analysis, which will help to obtain diagnostic information more quickly and accurately, hopefully replacing traditional measurement methods to optimize clinical flow.

## Data availability statement

The raw data supporting the conclusions of this article will be made available by the authors, without undue reservation.

## Ethics statement

The studies involving human participants were reviewed and approved by Ethics Committee of The First Affiliated Hospital of Shantou University Medical College. The patients/participants provided their written informed consent to participate in this study.

## Author contributions

BW and ZZ: conceptualization and design. YD: design the workflow, perform the data curation, and draft the manuscript. PC: image acquisition. LZ, YC, XC, and SJ: modify the manuscript. BW: project administration and funding acquisition. All authors revised the drafted manuscript, contributed to critical intellectual content, and read and agreed to the published version of the manuscript.
