# Supplementary material for: Myocardial strain analysis of echocardiography based on deep learning
Source: Front Cardiovasc Med. 2022 Dec 16;9:1067760. doi: 10.3389/fcvm.2022.1067760 (PMC9800889; doi:10.3389/fcvm.2022.1067760)
Supplement: Supplementary file 1 [file Data_Sheet_1.docx]

Supplementary Material

**Section 1. Architecture Comparison**

The architecture comparisons of RAFT, Pwc-net, and Flownet (**Supplementary Figure 1**): The core idea of the three network architectures is to extract features from adjacent images, calculate correlation on feature layer and estimate optical flow field. The main difference is the flow field update process. RAFT adopts lightweight recurrent neural network to optimize the flow field iteratively. The number of cycles is unlimited and can be set by the author according to the actual situation. While, Pwc-net adopts the structure of spatial pyramid to optimize the flow field. The initial flow field is optimized from bottom to top, and the iteration times are at most 6 times, limited by the number of pyramid layers. Optical flow from coarse to fine. Different from the above multiple refinement strategy, Flownet is similar to encoder-decoder architectures with image warp and propagation of brightness error. Encoder extracts image features and generates optical flow directly through decoder pixel restoration.


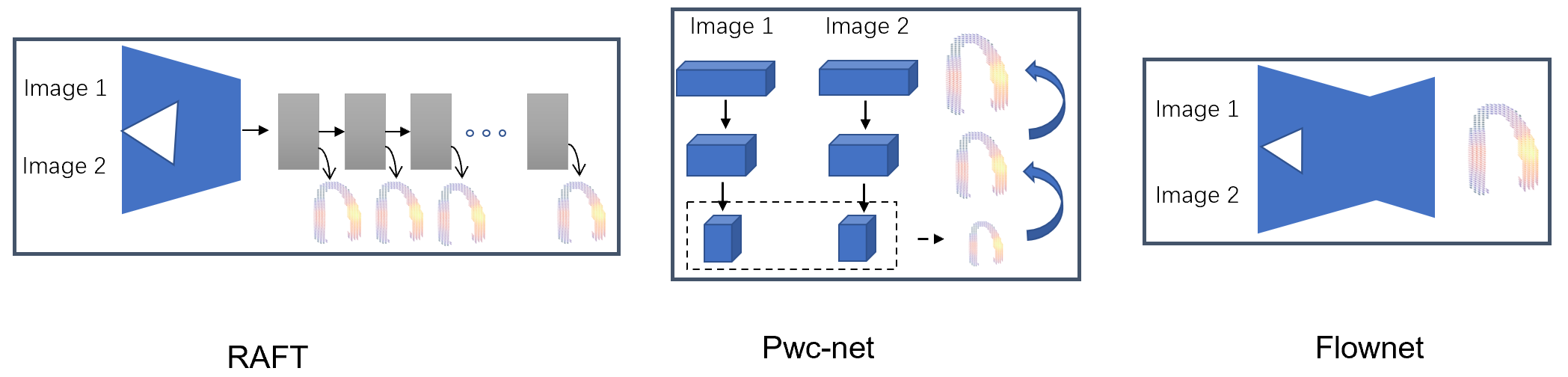


**Supplementary Figure 1. A schematic diagram of the network structure.** It shows the different refinement processes of images after feature extraction

**Section 2. Data Augmentation**

For the robust of the model, general data augmentation such as horizontal and vertical flipping, random cropping, scaling, and video clipping were used. In order to diversify the forms of movement fields between frames, we also adopted the method of compartment frame taking and time reversal to generate optical flow ground-truth. Each cardiac cycle of the simulated echocardiogram video contains 54 to 70 frames, which are high frame rate videos. But the actual echocardiogram one cardiac cycle frame number distribution is 20-30. Considering the influence of different FPS on the motion amplitude between frames. We extracted images at random intervals of one or two frames to generate the movement fields between them, and reversely calculated the movement fields between frames as the ground-truth of optical flow, so as to expand the distribution range of sports fields. Furthermore, we fixed the simulated video at 30 frames through directly clips and sampling every other frame, thus each raw video was cut into 4 cine-loop which represent different amplitude of motion between frames. In the end, 105 videos were edited into 420 videos, each containing 30 frames. Each video was further cut into 10 three-frame linked data for segmentation training and 29 image pairs for motion network training.Finally, 4200 fully labeled echocardiogram data were generated for 3D-CSN training, and 12,000 ultrasound image pairs for the development of the motion network. After augmentation, the flow field has a wider distribution range and is more consistent with clinical data. In addition, ultrasound-specific augmentation routines were applied, including depth attenuation, haze artifact application and gaussian shadowing (**Supplementary Figure 2**). Except for video clipping, data augmentation is done on-the-fly, which results in as many different images as training iterations.

**Depth attenuation:** The ultrasound wave looses energy as it travels through the body, and this can be identified as a gradual drop in intensity with distance from the probe. Similarly like the haze artifact application, we apply a varying degree of intensity attenuation along the radial direction. The attenuation does not consider depth independent noise, and is thus a simplification of the physical artifact.

**Haze artifact application:** One artifact that is prevalent for some patient is acoustic haze. This can be identified as a semi-static noise band in the upper parts of the image. We randomly apply static high intensity artifacts with a gaussian profile along the radial direction in polar coordinates.

**Shadowing:** Acoustic shadows often occur in ultrasound imaging due to structures that strongly reflect or absorb the ultrasound waves. This is often identified as a dark region behind the structure. We mimic this effect by placing random regions of intensity reductions in the image. Similar methods have been shown to have an effect on generalization for ultrasound segmentation tasks.


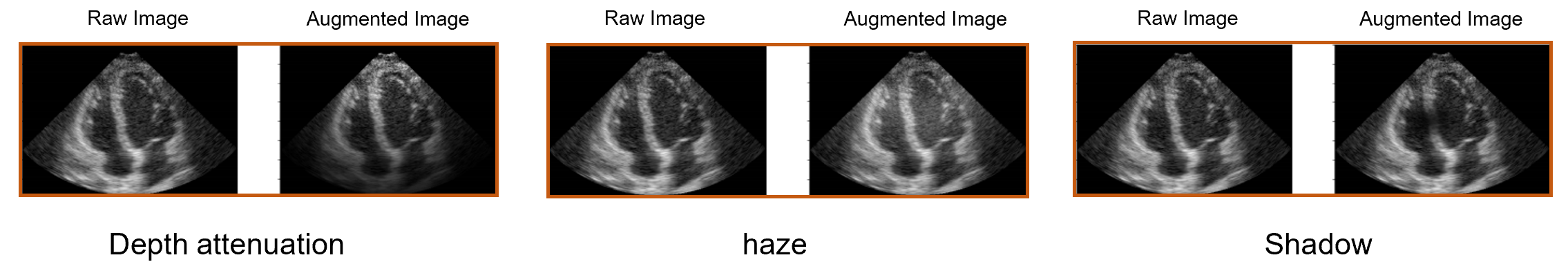


**Supplementary Figure 2. Ultrasound-specific augmentation.** From left to right, the raw image and the augmented image are displayed respectively.

**Section 3. Acquisition Criterion**

To achieve a study population with a wide range of cardiac function and different pathologies, we included 3 pre-defined patient groups: patients with myocardial infarction (MI), patients with heart failure (HF), and patients admitted for chest pain without any evidence of cardiac origin.

**MI was defined according to**: any of the following: 1) Increased and/or decreased cardiac biomarker (preferred cTn) with at least one value exceeding the upper 99th percentile of the reference limit and at least one evidence of myocardial ischemia: symptoms of ischemia, ECG changes suggestive of new ischemia (new ST-T changes or new LBBB), pathological Q wave formation, and radiographic evidence of new viable myocardial loss or new regional ventricular wall movement abnormalities; 2) Pathological features of acute myocardial infarction or myocardial infarction in healed or healed stage.

**HF was defined according to**: Present or pre-existing: Signs and symptoms of heart failure due to heart structure and/or function (including EF<50%, heart enlargement, E/E '>15, moderate/severe ventricular hypertrophy, moderate/severe cardiac valve stenosis or regurgitation); Simultaneously combine at least one of the following items: 1) Increased natriuretic peptide level; 2) Objective evidence of cardiogenic pulmonary or systemic circulation congestion, including imaging (e.g., chest radiography, echocardiography, etc.), resting or stress (e.g., exercise) hemodynamic monitoring (e.g., right heart catheter, pulmonary artery catheter, etc.), etc.

**Patients with chest pain without cardiac origin**, including neither clinical examination, laboratory tests, electrocardiogram (ECG), echocardiography, or coronary angiography revealed any evidence of possible cardiac origin of chest pain.

**Supplementary Tables**

SUPPLEMENTARY TABLE 1 | Summary of characteristics of clinically validated patients

| Characteristics | Total (50) |
| --- | --- |
| Study cohorts  Myocardial infarction  Ischemic heart failure  No significant cardiac disease | 17 (34%)  17 (34%)  16 (32%) |
| Demographics  Age, years (SD)  Male, n (%)  Diabetes Mellitus, n (%)  Hypertension, n (%) | 63±17  35 (70%)  8 (16%)  36 (72%) |
| Study cohorts  Myocardial infarction  Ischemic heart failure  No significant cardiac disease | 17 (34%)  17 (34%)  16 (32%) |
| Echocardiographic measurements  LVEF, %  LVEDV, ml  LVESV, ml | 46±13  103±76  67±61 |
| LV function by LVEF category  Severely reduced: <30%  Moderately reduced: 30-39%  Mildly reduced: 40-49%  Normal: >50% | 7 (14%)  8 (16%)  12 (24%)  23 (46%) |

SUPPLEMENTARY TABLE 2 | Intra-group consistency comparison (ICC) of regional longitudinal strain of test set

| Region | ICC [95%CI] |
| --- | --- |
| Basal anterolateral | **0.85** **[0.26-0.98]** |
| Basal anteroseptal | 0.44 [-1.35-0.70] |
| Basal inferior | 0.56 [-0.26-0.94] |
| Basal anterior | 0.44 [-0.38-0.70] |
| Basal inferolateral | 0.61 [0.08-0.82] |
| Basal inferoseptal | 0.76 [0.25-0.92] |
| Mid anteroseptal | 0.60 [-0.16-0.95] |
| Mid anterolateral | **0.90** **[0.43-0.99]** |
| Mid inferior | 0.74 [0.23-0.89] |
| Mid anterior | 0.71 [-0.10-0.97] |
| Mid inferolateral | 0.62 [0.03-0.85] |
| Mid inferoseptal | 0.41 [-0.93-0.89] |
| Apical spetal | 0.31 [-1.21-0.85] |
| Apical inferior | 0.57 [0.10-0.70] |
| Apical lateral | 0.64 [-0.19-0.95] |
| Apical anterior | **0.83 [-0.03-0.98]** |


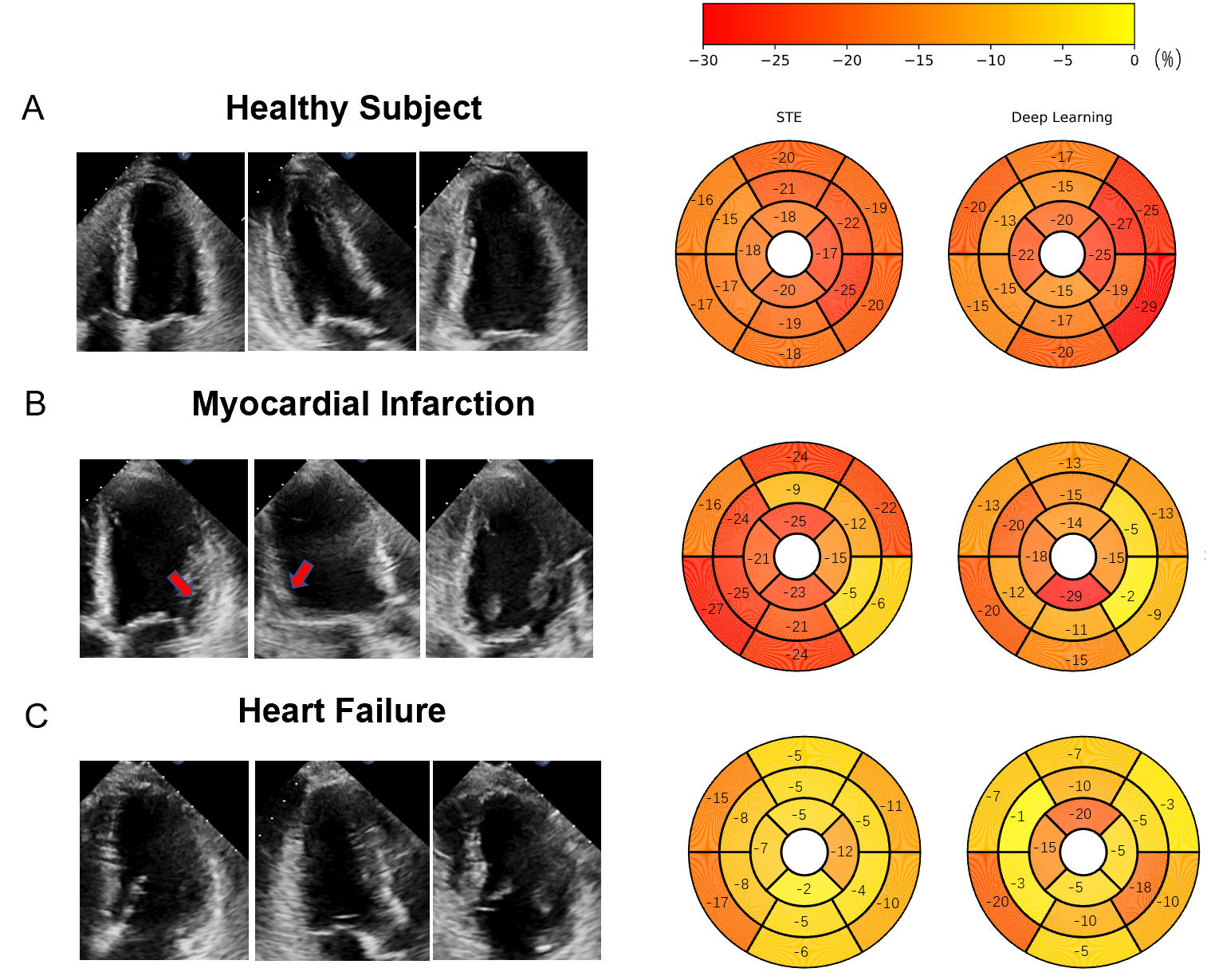


**Supplementary Figure 3**：Example of differences in longitudinal regional strain estimates by different modalities. (A), Echocardiograms and bull’s eye maps of healthy subject, strain is homogenously distributed. (B), MI subject shows a focal strain reduction in the inferolateral region co-localized with the infarcted region (red arrows). (C), HF subject shows diffused strain reduction.
